# Supplementary material for: Noncontact Gesture-Based Switch Improves Communication Speed and Social Function in Advanced Duchenne Muscular Dystrophy: A Case Report
Source: Healthcare (Basel). 2025 Nov 20;13(22):2989. doi: 10.3390/healthcare13222989 (PMC12652835; doi:10.3390/healthcare13222989)
Supplement: Supplementary file 1 [file healthcare-13-02989-s001.zip › healthcare-3963474-supplementary.pdf]

Supplementary Table S1. SF-36 each items. Note: PF items (Q3–12) were missing and therefore not applicable.

Item No.	Question (summary)	Pre	Post
1	Current health status	2	2
2	Health compared to 1 year ago	2	2
3–12	Physical functioning (PF10 items)	—	—
13	Reduced time on work/regular activities (RP1)	5	5
14	Accomplished less than desired (RP2)	5	5
15	Could not do some activities due to their nature (RP3)	5	5
16	Had difficulty performing work/activities (extra effort) (RP4)	5	5
17	Reduced time due to emotional problems (RE1)	5	5
18	Accomplished less due to emotional problems (RE2)	5	5
19	Did not perform work/activities as carefully as usual (RE3)	5	5
20	Extent health/emotional problems interfered with social activities (SF1)	1	1
21	Bodily pain intensity (BP1)	1	1
22	Pain interference with normal work (BP2)	1	1
23	Felt full of pep (VT1)	2	2
24	Felt nervous (MH1)	5	5
25	Felt downhearted and blue (MH2)	5	5
26	Felt calm and peaceful (MH3)	2	2
27	Had energy (VT2)	3	3
28	Felt down/depressed (MH4)	5	5
29	Felt worn out (VT3)	5	5
30	Felt happy (MH5)	1	2
31	Felt tired (VT4)	3	3
32	Social activity limitation due to health/emotional problems (SF2)	5	5
33	I get sick easier than others (GH1)	5	5
34	I am as healthy as anyone I know (GH2)	2	2
35	I expect my health to get worse (GH3)	5	5
36	My health is excellent (GH4)	2	2
